# Interpreting rare missense variants in *ABCB4* using ACMG recommended in silico tools, bespoke prediction algorithm Vasor and AlphaMissense.

**DOI:** 10.1038/s41598-026-49993-z

**Published:** 2026-05-02

**Authors:** Alexander Bracanovic, Catherine Williamson, Julia Zöllner, Peter H. Dixon

**Affiliations:** 1 Department of Women and Children’s Health, King’s College London, Guy’s Campus, London, UK; 2 Institute of Reproductive and Developmental Biology, Imperial College London, Hammersmith Campus, London, UK; 3 Institute for Women’s Health, University College London, London, UK

**Keywords:** ICP, ABCB4, ACMG, AlphaMissense, Computational biology and bioinformatics, Diseases, Genetics

## Abstract

Intrahepatic Cholestasis of Pregnancy (ICP) is the most common pregnancy-related liver disorder. *ABCB4* heterozygous variants are implicated in ICP, but interpretation of rare variants remains challenging, leading to many variants of uncertain significance. This study compares predictions from multiple in silico tools on nine heterozygous missense variants identified in women with ICP. Using the Genomics England Research Environment, 253 women with ICP and whole-genome sequencing data were analysed. Variants with minor allele frequency < 0.05 were filtered, and nine rare missense variants were assessed using SIFT, PolyPhen, CADD, Vasor (*ABCB4*-specific), and AlphaMissense. Only 44% (4/9) of variants had consistent classification across all tools. Published functional studies often conflicted with predictions. For example, T175A showed no detectable effect in HepG2/HEK293 cells but was classified as likely pathogenic by Vasor and benign by AlphaMissense. Similarly, N510S impacted protein stability functionally and was deemed likely pathogenic by Vasor but benign by AlphaMissense. In silico tools show conflicting predictions for *ABCB4* rare variants, highlighting the difficulty of classification without functional or segregation data as well as the heavy reliance on computational predictions.

**Supplementary Information:**

The online version contains supplementary material available at 10.1038/s41598-026-49993-z.

## Introduction

Cholestatic liver disorders are characterised by insufficient clearance of bile salts, resulting in liver damage and fibrosis. These conditions encompass a range of diseases, including Progressive Familial Intrahepatic Cholestasis (PFIC), Intrahepatic Cholestasis of Pregnancy (ICP), and Low-Phospholipid-Associated Cholelithiasis (LPAC)^1^. Understanding the genetic underpinnings of these disorders is crucial for elucidating their pathogenesis and developing targeted therapies.

Numerous genetic variants across various genes have been associated with cholestasis. Notably, variants in the ATP-binding cassette 4 (*ABCB4*) gene have been implicated in several cholestatic disorders, underscoring their role in disease mechanism and pathogenesis^2^.

*ABCB4*, located on chromosome 7q21.1, has 27 exons and spans approximately 81 kb of genomic sequence. MDR3, the protein product of *ABCB4*, plays a critical role in translocation of phosphatidyl choline from the inner to outer layer of the hepatocyte canalicular membrane for clearance by bile salts^3^. These phospholipids are essential for the formation of mixed micelles, which prevent the precipitation of cholesterol and protect the biliary epithelium from bile salt toxicity. When micelle formation is compromised due to low phospholipid levels in the bile, the relatively high concentration of bile acids in the bile can result in cholangiopathy. Thus, genetic variants in *ABCB4* can significantly impact the function of MDR3, thereby exacerbating bile salt toxicity and contributing to liver disease^4^. Pathogenic rare variants in *ABCB4* have been implicated in up to 20% of patients with severe ICP^5,6^. Low-phospholipid-associated cholelithiasis (LPAC) is another disorder associated with *ABCB4* variants; the reduced phosphatidylcholine secretion leads to the formation of gallstones in the liver and gallbladder^7^.

Intrahepatic Cholestasis of Pregnancy (ICP) is the most common pregnancy related liver disorder and typically resolves rapidly postpartum. Maternal clinical manifestations include pruritus, dark urine, pale stool and in rare cases jaundice^8^. The associated elevation in serum bile acid concentrations is associated with increased risk of spontaneous preterm birth and stillbirth^9,10^. The genetic architecture of ICP can be categorized as complex with common and rare variants in *ABCB4* contributing to the aetiology of ICP and related phenotypes^4,2,5,11^.

### In Silico computational and predictive data

The American College of Medical Genetics and Genomics (ACMG) provides the most widely clinically adopted framework for variant interpretation, incorporating several criteria against which variants are evaluated^12^. These criteria primarily include the use of population data, computational data, and functional data. Whilst each criterion is assessed separately during variant interpretation, an aggregation of complementary scores from each category is necessary to achieve a conclusive variant classification. For rare missense variants, the absence of functional studies places a significant burden on computational predictive data which may not always be accurate enough to predict functional effect^13^. The ACMG recommends utilising as many predictive tools as feasible, favouring those with diverse algorithmic bases for prediction. Additionally, when computational data is used as a criterion for classification (e.g., PP3 for pathogenic and BP4 for benign), it is essential that all predictors agree on the variant effect.

In this study, we evaluated the utility of commonly used in-silico prediction tools for rare *ABCB4* missense variant interpretation in the context of intrahepatic cholestasis of pregnancy. We selected *ABCB4* as a clinically relevant case study because rare missense variants in this gene are implicated in ICP and related cholestatic phenotypes, yet many remain difficult to interpret confidently in the absence of segregation or functional evidence. Our aim was not to propose a gene-specific classification framework, but to assess how computational predictors perform in a disease-relevant, gene-focused setting and to examine the extent to which their outputs align with available clinical and functional evidence.

## Methods

### Cohort curation

The Genomics England Research Environment (GEL-RE) offers a comprehensive resource of whole genome sequencing (WGS) data for genomic studies^14^. High depth WGS generates low levels of sequence missingness, allowing for superior identification of rare genetic variants. The GEL-RE was utilised to call variants in an ICP case cohort. To accurately identify patients who had been previously diagnosed with ICP, ICD-10 phenotype coding was used, O26.6, liver disorders in pregnancy. Our cohort was curated using the Genomics England application programming interface (API) software, R LabKey API, run in RStudio 4.2.1. Informed consent was obtained from all participants by Genomics England prior to inclusion in the study. All methods were carried out in accordance with relevant guidelines and regulations and were reviewed and approved by Genomics England and its Ethics Advisory Committee (REC reference 14/EE/1112).

### Variant calling and filtering

To gain an initial comprehensive overview of all variants identified in *ABCB4*, the Genomics England Small Variant Workflow was implemented in the high-performance cluster (HPC). The workflow was run using a Nextflow DSL1 script which included containerisation of R, Python, bcftools and VEP. The output of this workflow was filtered by including all missense variants which had a cohort minor allele frequency (MAF) of < 0.05.

### In Silico implementation

Variants were tested across 5 in silico tools, three of which, SIFT^15^, Polyphen-2^16^ and CADD^17^, were ACMG cited for use in variant classification. The ACMG guidelines was the sole framework for informing use of in silico tools in variant classification. Vasor^18^, a bespoke prediction tool for *ABCB4* missense variants as well as AlphaMissense^19^, a novel variant effect predictor, were also included. In silico predictions for all except Vasor were generated through Ensembl VEP v112 web interface^20^. A diverse set of algorithmic bases were chosen in order to satisfy the ACMG’s recommendation for PP3/BP4 and are described in Table 1.

**Table 1:** In Silico tool classification thresholds and algorithmic basis.

In Silico Tool	Classification Threshold	Algorithmic Basis
SIFT	Scores ≤ 0.05 were classified as deleterious	Sequence conservation, multiple sequence alignment, and amino-acid substitution probabilities.
PolyPhen-2 (HumVar)	Scores > 0.85 were considered probably damaging, 0.15–0.85 possibly damaging and < 0.15 benign	Supervised classification using sequence-based and structure-based features.
CADD v1.6 PHRED	Scores ≥ 20 were interpreted as supportive of pathogenicity	Integrated, genome-wide score trained to distinguish simulated de novo variants from human reference; incorporates conservation, regulatory, and protein-level features.
Vasor (Access date: 25/08/2025)	Vasor probabilities $\:\ge\:0.5$ were considered likely pathogenic	Gene-specific supervised model for *ABCB4* using curated functional data and diverse structural, conservation and physicochemical features.
AlphaMissense	Scores ≥ 0.56 were interpreted as pathogenic, ≤ 0.34 benign with intermediate scores being classed ambiguous.	Deep learning model leveraging AlphaFold structure-based features and evolutionary information without direct use of gene-specific prior classifications.

### In Silico performance analysis

#### ClinVar-labelled *ABCB4* missense variants

To determine the accuracy of the predictive tools in their ability to classify *ABCB4* missense variants, ClinVar^21^ was used as a search tool and benchmark for pathogenic or benign clinical significance. Variants with “pathogenic/likely pathogenic” ClinVar reports were grouped as pathogenic whilst those with benign/likely benign were grouped as benign. Those with conflicting, uncertain or missing interpretations were excluded. The date of last evaluation for all ClinVar variant classifications used were ensured to be post 2015 to reflect the timing of the ACMG/AMP variant interpretation guideline publication. Allele frequency was not a component of the filtering criteria to both maximise sample size whilst also considering the genetic complexity of cholestatic liver disorders like ICP, which have both a common and rare variant effect contribution. A confusion matrix was determined for each of the in silico tools in comparison to their respective benchmark.

### Comparative test statistics

Matthews Correlation Coefficient (MCC) was calculated to determine correlation between predicted and observed classification. A positive coefficient closer to 1 suggests strong predictive ability. A coefficient closer to 0 on the other hand, suggests that the predictor is no better than a random model.

$$\:MCC=\:\frac{\left(TP\times\:TN\right)-(FP\times\:FN)}{\sqrt{(TP+FP)(TP+FN)(TN+FP)(TN+FN)}}$$

F1 was calculated to determine mean precision and recall. These are defined respectively as the proportion of positive predictions that are true positives and the number of true positives that were accurately predicted. F1 is scored from 0 to 1 where 1 indicates perfect precision and recall.

$$\:Precision=\frac{TP}{TP+FP}$$

$$\:Recall=\:\frac{TP}{TP+FN}$$

$$\:F1=2\times\:\frac{\:Precision\times\:Recall}{Precision+Recall}$$

## Results

### Cohort curation

Curation of the ICP cohort using the LabKey API through SQL queries in R initially yielded 284 participants. After removal of duplicate participant entries and search for available corresponding Variant Call Format (VCF) files pertaining to these participants the final cohort included in analysis was 253. Overall, this cohort was predominantly of self-declared white ethnicity (*n* = 193).

### Variant calling

**Fig. 1:**
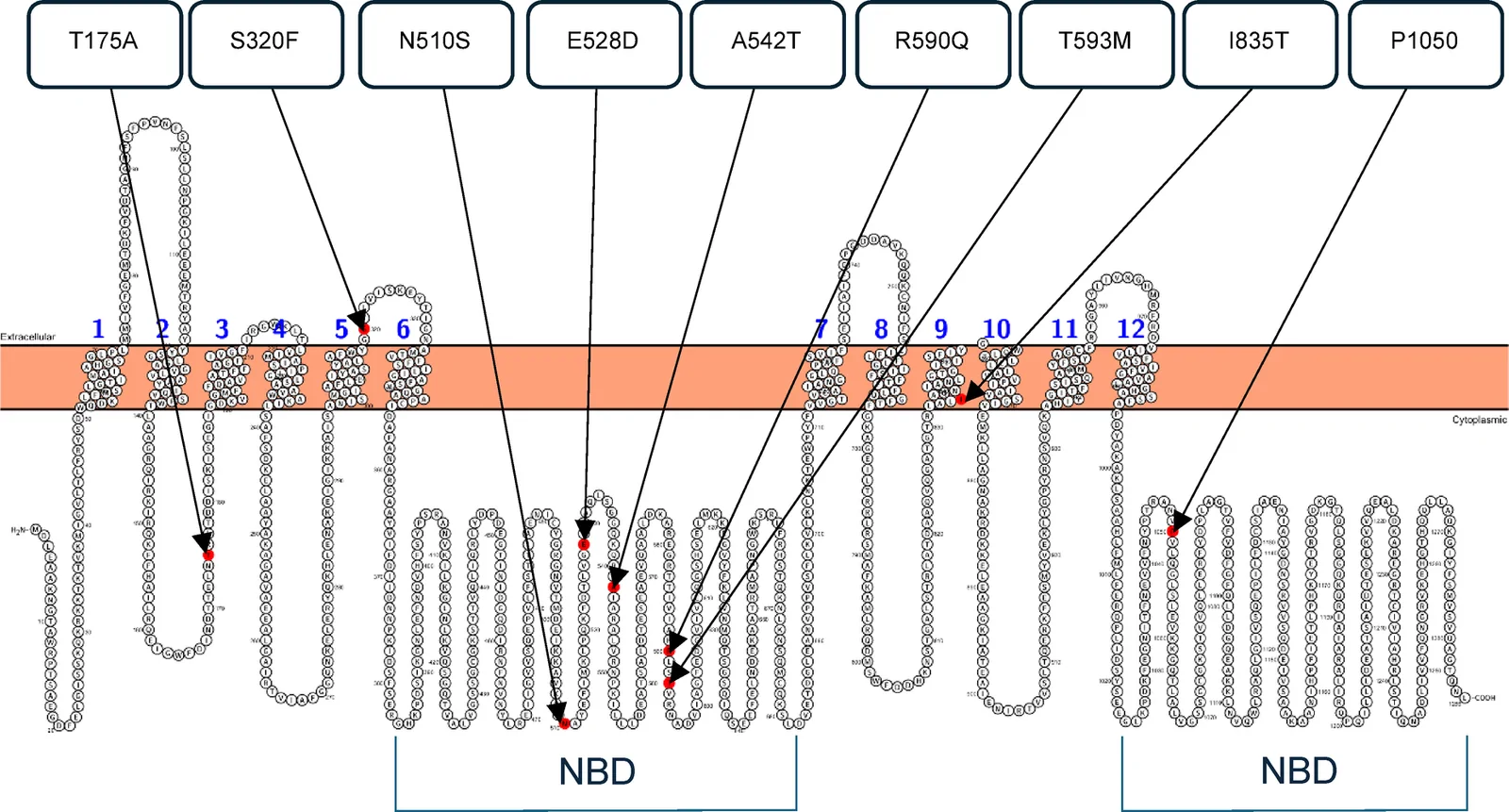
Topographic representation of ABCB4 and amino acid changes from rare missense variants identified. Nucleotide Binding Domain (NBD) 1 (*n* = 5), NBD2 (*n* = 1), Transmembrane Domain (TMD) 2–3 (*n* = 1), TMD 5–6 (*n* = 1), TMD9 (*n* = 1)^23^.

Post filtering for non-synonymous variants with a MAF < 0.05 in transcript ENST00000649586, a total of 18 variants were identified. Splice variants (*n* = 3), frameshift variants (*n* = 4) and nonsense variants (*n* = 2) were excluded from analysis leaving only rare missense variants. Of the 9 rare missense variants identified in our ICP cohort, all were heterozygous, and one was novel, chr 7. 87,408,168 G > A p. P1050S, which was recently cited in a study investigating rare *ABCB4* variants in ICP^5^. As seen in Fig. 1, most missense variants identified were in nucleotide binding domain (NBD) 1 *n* = 5. One was identified in NBD2 and transmembrane domain (TMD) 9. Two variants were found between TMDs one being in a cytoplasmic region and the other in an extracellular region.

### In Silico Implementation

As shown in Table 2 only 44% (4/9) of variants had full concordance over classification into pathogenic or benign across all prediction tools. Consequently, ACMG PP3 or BP4 could only be applied to those variants, with the remaining and majority of variants at a higher likelihood of an overall classification of uncertain significance. No difference between prediction and protein domain was observed.

**Table 2:** Missense Variants identified with their respective in silico predictions. Red boxes indicate a pathogenic prediction whilst green boxes indicate a benign prediction. Yellow box represents ambiguous.

*Genomic Position*	*Protein Change*	*GnomAD Population Frequency*	*Domain of Amino Acid Change*	*SIFT*	*POLYPHEN-2*	*CADD PHRED*	*Vasor*	*AlphaMissense*	*ACMG PP3/BP4*
chr7: 87,452,957 T > C	T175A	0.009335	TMD2 −3	0 (deleterious)	0.625 (possibly damaging)	23.9	0.597158194 (likely pathogenic)	0.1388 (benign)	✖
chr7: 87,447,080 G > A	S320F	0.0001735	TMD5–6	0 (deleterious)	0.852 (possibly damaging)	26.4	0.745021999 (likely pathogenic)	0.6872 (pathogenic)	✔
chr7: 87,440,230 T > C	N510S	0.0001927	NBD1	0 (deleterious)	0.859 (possibly damaging)	23.8	0.876476526 (likely pathogenic)	0.1281 (benign)	✖
chr7: 87,439,814 C > G	E528D	0.001514	NBD1	0.24 (tolerated)	0.017 (benign)	3.616	0.251453936 (likely benign)	0.1229 (benign)	✔
chr7: 87,439,774 C > T	A542T	0.000002478	NBD1	0.01 (deleterious)	0.99 (probably damaging)	24.6	0.896902978 (likely pathogenic)	0.5798 (pathogenic)	✔
chr7: 87,431,528 C > T	R590Q	0.006699	NBD1	0 (deleterious)	0.967 (probably damaging)	27.8	0.759965003 (likely pathogenic)	0.8113 (pathogenic)	✔
chr7: 87,431,519 G > A	T593M	0.00002726	NBD1	0 (deleterious)	1 (probably damaging)	27.8	0.900535405 (likely pathogenic)	0.4254 (ambiguous)	✖
chr7: 87,417,490 A > G	I835T	0.000006195	TMD9	0.01 (deleterious)	0.755 (possibly damaging)	26.2	0.204421818 (likely benign)	0.2612 (benign)	✖
chr7: 87,408,168 G > A	P1050S	0.0000006195	NBD2	0.21 (tolerated)	0.567 (possibly damaging)	23.9	0.220460773 (likely benign)	0.1336 (benign)	✖

### In Silico performance analysis

**Fig. 2:**
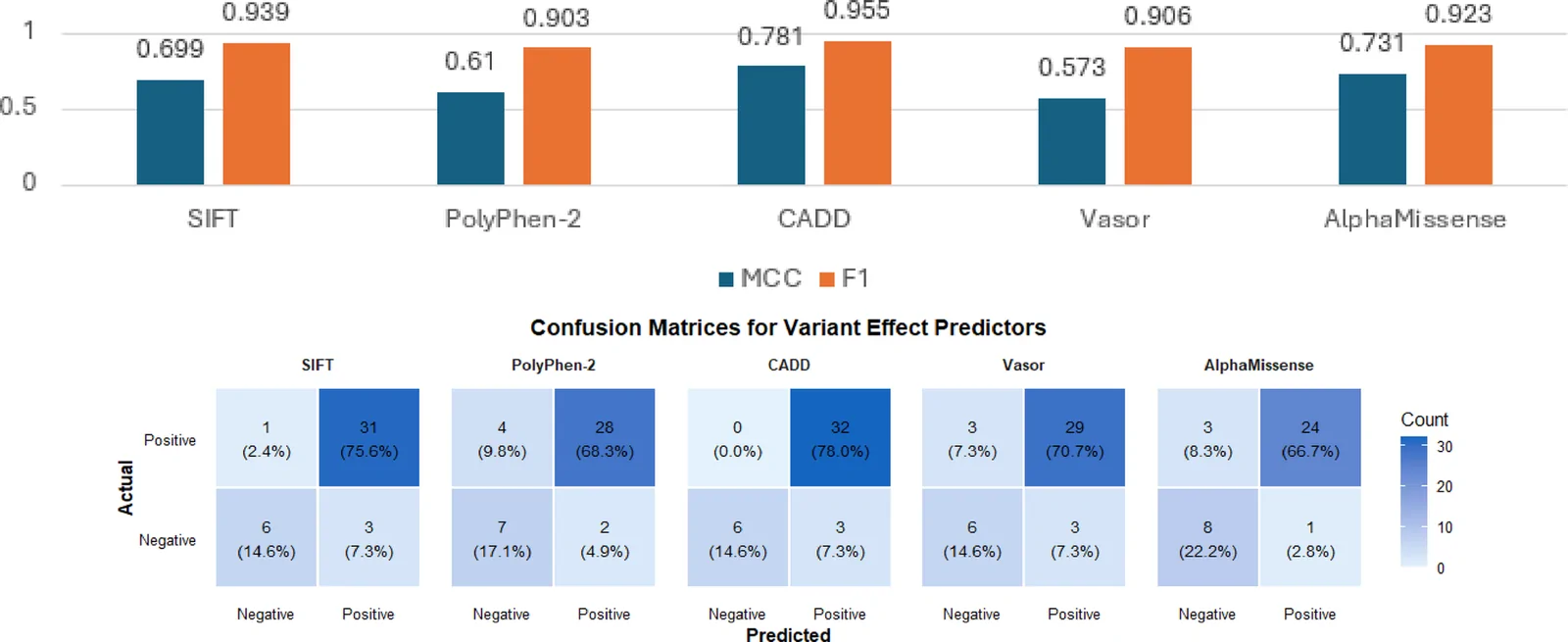
MCC and F1 of SIFT, PolyPhen-2, CADD, Vasor and AlphaMissense in broad ABCB4 Analysis. The best performing tool was CADD with an F1 of 0.955 and an MCC of 0.781, closely followed by AlphaMissense with an F1 of 0.923 and MCC of 0.731. Confusion matrices are shown below for each tool.

In ClinVar, 41 missense variants with reliable labels were identified across *ABCB4*. From these, 3 were identified in our ICP cohort, 2 of which were both classified and benchmarked as benign and 1 as pathogenic. As shown in Figure 2, in this dataset, CADD and AlphaMissense achieved the highest performance with MCC of 0.781 and 0.731 respectively and $\:{F}_{1}$ scores of 0.955 and 0.923. The lowest performing tools in this dataset were Vasor and PolyPhen-2 with similar $\:{F}_{1}$ scores of 0.906 and 0.903 each, and MCC scores of 0.573 and 0.6. Similarly to the ICP variant score performance analysis, the datasets were slightly unbalanced with AlphaMissense using 36 variants whilst the other tools used 41.

## Discussion

The gene-specific focus of this study is relevant because *ABCB4* encodes the canalicular phosphatidylcholine transporter MDR3; disruption of this function has direct mechanistic consequences for bile composition and cholestatic injury^24^. The diverse functional impact of different *ABCB4* variants poses a significant diagnostic challenge, as mutations can lead to a broad spectrum of hepatobiliary disorders. Consequently, clinical presentation is often inconsistent, and individuals with the same *ABCB4* genotype may display markedly different clinical phenotypes^25^.

In our ICP cohort, for the variants that were concordantly predicted to be pathogenic, several structural considerations may explain their predicted deleterious effects. Variants located within the NBDs are likely to disrupt conserved motifs required for ATP binding and hydrolysis, such as the Walker A/B motif^26^. In contrast, variants situated within the TMDs may interfere with substrate translocation^27^. Given the structural and functional complexity of transmembrane proteins such as *ABCB4*, in silico tools may have reduced accuracy in distinguishing between variants that primarily affect catalytic activity, substrate transport or protein stability, underscoring the need for functional validation to clarify their precise molecular consequences.

For the majority of *ABCB4* variants identified in our ICP cohort, computational evidence was ineligible for use as support for classification. Although there was strong concordance between SIFT, PolyPhen-2, and CADD, with the only discrepancy observed being with the novel P1050S variant, predictions from Vasor and AlphaMissense were less consistent. Furthermore, a review of published functional studies revealed additional inconsistencies in reported findings^28,29^. This was particularly true for predictions from AlphaMissense, which reports higher prediction accuracy than conventional tools, and Vasor, a tool specifically developed for the interpretation of missense *ABCB4* variants.

In a functional study investigating *ABCB4* variants contributing to severe, early-onset, progressive cholestasis, HepG2 and HEK293 cell lines were transfected with plasmids encoding either *ABCB4* wild type or disease-causing variants. In that study, two variants identified in our cohort, T175A and N510S, were classified based on their impact on protein maturation, activity, stability, or as having no detectable effect. T175A was classed as having no detectable effect whilst N510S was classed as impacting protein stability and reducing protein levels compared to the wild type despite resulting in a fully processed and active *ABCB4* protein^28^. In our study, Vasor predicted T175A as likely pathogenic whilst AlphaMissense classified it as benign. Conversely, N510S was classified as a likely pathogenic by Vasor, aligning with the functional study results. However, AlphaMissense provided a benign interpretation for the same variant in our study, indicating a discrepancy. Separately, uncertainty was identified around one of the unanimously pathogenic predicted variants by all in silico tools. In a study by Pauli-Magnus et al. (2004), R590Q was one of the few identified rare *ABCB4* variants found only in a healthy control cohort (*n* = 149) when identifying variants in patients that had been diagnosed with either primary biliary cirrhosis (*n* = 76) or primary sclerosing cholangitis (*n* = 46). Whilst this variant was not formally classified, its absence in cases and presence in controls suggests that its impact on *ABCB4* is likely more complex than is suggested by the in silico predictions^29^.

Clinically, these findings are relevant because rare *ABCB4* missense variants are frequently encountered in the evaluation of ICP and related cholestatic phenotypes, yet many remain classified as variants of uncertain significance. Our results indicate that computational evidence can support interpretation in some cases, but disagreement among predictors remains common even within a single clinically important gene. This reinforces the need to apply PP3/BP4 evidence cautiously and to avoid overinterpreting concordant or discordant in-silico predictions in the absence of additional data. For patients with suspected ABCB4-related disease, clinically meaningful interpretation will often require integration of computational outputs with phenotype, segregation, population frequency, and, where available, functional evidence.

To attempt to quantify the accuracy of individual predictors, our in silico performance analyses showed differences based on assessment method, highlighting the strengths and limitations of these tools in classifying *ABCB4* missense variants. Two sperate accuracy calculations were used to compare performance, MCC which places equal value to identification of true positives and true negatives and $\:{F}_{1}$ which determines performance based on increased weight on true positives. An MCC score can only be high if the predictor performs well with both positive and negative values. Our performance analysis including all *ABCB4* missense variants with a conclusive pathogenicity classification in ClinVar. A clear distinction in performance metrics was seen in CADD and AlphaMissense with a marked reduction in performance seen in Vasor. Variants of uncertain significance still pose a significant challenge in variant interpretation and as such, this demonstrates the potential utility of using AlphaMissense to evaluate hard to classify variants. The results obtained in our evaluation, particularly of AlphaMissense, have been similarly reflected in a recent study which evaluated its performance in 1,500 variants across 42 genes encoding ABC proteins (MCC of 0.646 and a F1 score of 0.850)^30^. Future work should aim to continue to improve benchmarking and validation by creating larger functional datasets and incorporating new protein language models such as ESM-2 to improve missense variant prediction accuracy^31^.There were several limitations to this work, mainly driven by the limited numbers of variants in our study and benchmarking suitability. Whilst ClinVar is often used as a standard for functional reporting and interpretation of variant effect, it has previously been suggested the use of a bespoke gene specific database can be more beneficial. This was highlighted in a previous study which found that AlphaMissense demonstrated stronger performance for classifying *CFTR* (an ABC protein coding gene) variants when benchmarked against the higher quality CFTR2 database as opposed to ClinVar^30^. This difference in benchmark quality is likely due to resources such as ClinVar leveraging computational predictions as a component of their variant classifications, resulting in a degree of circularity when used to assess individual tools. As such, our results should only act as case sensitive guidelines for *ABCB4* variant classification performance. Furthermore, a recommendation from the ClinGen Sequence Variant Interpretation Working Group has suggested that using one tool, which has well calibrated thresholds through the implementation of Bayesian statistical techniques with functional data from variant curation expert panels, can be more insightful than using multiple which have not been validated in this way^32^. The paucity of clinical and functional data in the 9 rare missense variants identified in our ICP cohort provided a crucial challenge when trying to determine which tools’ classifications were the most plausible. Consequently, further work, particularly in complex protein classes, such as ABC transporters, should be encouraged to provide additional supporting criteria for ACMG classifications as well as improved benchmarking for computational tools. A further limitation is the lack of an independent external validation dataset composed of expertly curated *ABCB4* missense variants with standardised classification and orthogonal evidence. Such a dataset would ideally include detailed phenotyping across *ABCB4*-associated cholestatic disorders, expert ACMG-based review, segregation information, and functional assays addressing expression, localization, stability, and phosphatidylcholine transport. Accordingly, the present findings should be generalised cautiously: they are most informative as an assessment of the behaviour and limitations of in-silico tools in an *ABCB4*-specific setting, rather than as a definitive estimate of clinical classification performance across all contexts. Finally, in our ICP cohort, most participants were of White European ancestry, limiting the generalisability of these variants in different populations. In future work, more should be done to encourage the participation and inclusion of people of diverse ethnic ancestries in genomic studies.

In summary, the strong performance of AlphaMissense, particularly in our ClinVar analysis and in previously published literature, is likely associated with its avoidance of leveraging prior knowledge to influence classifications. This is due to variants from the same gene often being jointly labelled as pathogenic or neutral based on known variant effect. Consequently, tools which predict pathogenicity based on this information struggle to accurately assess genes associated with disorders of lower incidence, for which few variants have been functionally assessed^33^. The performance of AlphaMissense, which predicts primarily based on protein structure change, has been shown in several instances to give different predictions compared to conventional tools. Its exclusion of prior knowledge to make assessments of variant pathogenicity mean that it may be able to provide alternative and novel insights for variants of unknown significance, which may prove important in identifying those with potentially genuine functional implications^34^.

## Summary

In conclusion, this study highlights the challenges of interpreting rare ABCB4 missense variants in the context of intrahepatic cholestasis of pregnancy and related cholestatic disease. By examining predictor performance in a clinically relevant, gene-specific setting, we show that in-silico outputs are often discordant and may not consistently reflect available functional or clinical evidence. These findings support the use of computational predictions only as one component of a broader interpretive framework and emphasize the continued need for well-curated datasets, functional validation, and careful clinical correlation in *ABCB4* variant assessment.

## Electronic Supplementary Material

Below is the link to the electronic supplementary material.

Supplementary Material 1

## Data Availability

Through the Genomics England secure Research Environment, approved researchers can access and analyse data in the National Genomic Research Library (NGRL). The NGRL is a repository of genomic and health data, including data from those participants recruited for the 100,000 Genomes Project, and from patients recruited and consented via the NHS Genomic Medicine Service (GMS). With ongoing recruitment and consent through the NHS GMS, Genomics England continuously add new clinical and genomic data to the NGRL, further enriching the data available for research. All data generated or analysed during this study are included in this published article [and its supplementary information files].

## Abbreviations

ABCB4: ATP-binding cassette 4
ACMG: American College of Medical Genetics and Genomics
API: Application programming interface
GEL-RE: Genomics England Research Environment
HPC: High-performance cluster
ICP: Intrahepatic cholestasis of pregnancy
LPAC: Low-Phospholipid-Associated Cholelithiasis
MAF: Minor allele frequency
MCC: Matthews Correlation Coefficient
NBD: Nucleotide binding domain
PFIC: Progressive Familial Intrahepatic Cholestasis
TMD: Transmembrane domain
WGS: Whole genome sequencing
